# Tolvaptan-induced hypernatremia related to low serum potassium level accompanying high blood pressure in patients with acute decompensated heart failure

**DOI:** 10.1186/s12872-020-01751-3

**Published:** 2020-10-29

**Authors:** Hidetada Fukuoka, Koichi Tachibana, Yukinori Shinoda, Tomoko Minamisaka, Hirooki Inui, Keisuke Ueno, Soki Inoue, Kentaro Mine, Kumpei Ueda, Shiro Hoshida

**Affiliations:** Department of Cardiovascular Medicine, Yao Municipal Hospital, 1-3-1 Ryuge-cho, Yao, Osaka 581-0069 Japan

**Keywords:** Acute decompensated heart failure, Hypernatremia, Hypokalemia, Tolvaptan

## Abstract

**Backgrounds:**

Tolvaptan significantly increases urine volume in acute decompensated heart failure (ADHF); serum sodium level increases due to aquaresis in almost all cases. We aimed to elucidate clinical factors associated with hypernatremia in ADHF patients treated with tolvaptan.

**Methods:**

We enrolled 117 ADHF patients treated with tolvaptan in addition to standard therapy. We examined differences in clinical factors at baseline between patients with and without hypernatremia in the initial three days of hospitalization.

**Results:**

Systolic (*p* = 0.045) and diastolic (*p* = 0.004) blood pressure, serum sodium level (*p* = 0.002), and negative water balance (*p* = 0.036) were significantly higher and serum potassium level (*p* = 0.026) was significantly lower on admission day in patients with hypernatremia (n = 22). In multivariate regression analysis, hypernatremia was associated with low serum potassium level (*p* = 0.034). Among patients with serum potassium level ≤ 3.8 mEq/L, the cutoff value obtained using receiver operating characteristic curve analysis, those with hypernatremia related to tolvaptan treatment showed significantly higher diastolic blood pressure on admission day (*p* = 0.004).

**Conclusion:**

In tolvaptan treatment combined with standard therapy in ADHF patients, serum potassium level ≤ 3.8 mEq/L may be a determinant factor for hypernatremia development. Among hypokalemic patients, those with higher diastolic blood pressure on admission may be carefully managed to prevent hypernatremia.

## Backgrounds

Tolvaptan, a selective V2 receptor antagonist with an aquaretic effect, significantly increases urine volume without increasing electrolyte excretion into the urine in acute decompensated heart failure (ADHF) [1–3]. Tolvaptan can decrease body weight, increase serum sodium level, and ameliorate some congestion symptoms in patients with ADHF, which may help prevent overdose of loop diuretics, especially in patients with renal dysfunction [4]. A meta-analysis of the published literature suggests short-term benefits of tolvaptan, but the impact on mortality is inconclusive [4–7]. The serum sodium level increases as a result of aquaresis in almost all cases, and hypernatremia can be lethal in some patients [8, 9] and was identified as a significant adverse event to be prevented [10]. Therefore, a lower dose of tolvaptan to prevent hypernatremia has been recommended in the initial phase [11, 12], because tolvaptan treatment can dose dependently lead to abnormal hypernatremia [13, 14]. Sometimes, hypernatremia results in central nervous system disturbance. There is a population that is a risk to the development of hypernatremia [15], and risk factors for hypernatremia in tolvaptan treatment were previously reported [10–12]. This study aimed to elucidate clinical factors associated with hypernatremia in patients with ADHF treated with full medications and tolvaptan in real-world practice.

## Methods

### Subjects

We retrospectively investigated 117 consecutive in-hospital patients with ADHF (mean age, 78 years) who received oral tolvaptan therapy in addition to standard therapy, including carperitide infusion, for the treatment of volume overload between January 2016 and December 2018 in our cardiology ward. Heart failure (HF) symptoms in all patients worsened despite treatment including oral diuretic therapy before hospital admission. Patients were excluded if they had anuria, consciousness disturbance, and cardiogenic shock.

### Procedure

All patients underwent baseline blood and urine tests, including neurohumoral assessment such as plasma B-type natriuretic peptide (BNP), renin activity, and aldosterone concentration, chest X-rays, and echocardiography on admission day. Serum osmolality was calculated using the following equation:

$${\text{Calculated}}\;{\text{serum}}\;{\text{osmolality}} = {2} \times {\text{Na}} + {\text{blood}}\;{\text{urea}}\;{\text{nitrogen}}/{2}.{8} + {\text{blood}}\;{\text{sugar}}/{18}.$$

Vital signs, 24-h fluid intake, and urine volume were measured at baseline and every 24 h thereafter. Body weight was measured after urination and before breakfast at baseline. First-morning spot urine tests included the measurements of osmolality and sodium (UNa), potassium, urea nitrogen (UUN), and creatinine (UCr) levels. The following formula was used to estimate urine osmolality:

$${\text{Urine}}\;{\text{osmolality }} = { 1}.0{7 } \times \, \left\{ {{2 } \times \, \left[ {{\text{UNa}}\;\left( {{\text{mEq}}/{\text{L}}} \right)} \right] \, + \, \left[ {{\text{UUN}}\;\left( {{\text{mg}}/{\text{dL}}} \right)} \right]/{2}.{8 } + \, \left[ {{\text{UCr}}\;\left( {{\text{mg}}/{\text{dL}}} \right)} \right] \, \times { 2}/{3}} \right\} \, + { 16}.$$

It was planned that all patients would undergo repeated blood and urine tests during 3 days after admission. Left ventricular ejection fraction was assessed by echocardiography using the biplane Simpson’s rule.

### Classification of hypernatremia

The development of hypernatremia was defined in a risk analysis when at least one measurement of serum sodium level was ≥ 148 mEq/L in the initial three days after tolvaptan treatment. Predictive factors that affect the development of hypernatremia by tolvaptan treatment were extracted from variables in clinical characteristics, blood and urine tests, and medications.

### Statistical analysis

All numerical data are expressed as mean ± standard deviation or percentages. Continuous data were compared using the unpaired t-test. Categorical data were assessed using the chi-square test. The area under the curve was calculated, and optimal cutoff values of predictors of hypernatremia were determined. A multivariate logistic regression analysis was applied to assess the independent factors showing hypernatremia using the variables that were significant in the univariate analysis. *p* values < 0.05 were considered statistically significant. All statistical analyses were performed using EZR (Saitama Medical Center, Jichi Medical University, Saitama, Japan), which is a graphical user interface for R (The R Foundation for Statistical Computing, Vienna, Austria).

## Results

### Baseline characteristics in patients with hypernatremia

Systolic (*p* = 0.045) and diastolic (*p* = 0.004) blood pressures were significantly higher on admission day in patients with hypernatremia (n = 22, Table 1). However, no differences were observed in comorbidities, such as diabetes, hypertension, and dyslipidemia, and medications before admission between patients with and without hypernatremia. The incidence of atrial fibrillation was also not different (Table 1). Regarding laboratory data, there were no differences in BNP level; estimated glomerular filtration rate; albumin, blood sugar, and uric acid levels; renin activity; and aldosterone level between the two groups (Table 1). However, serum sodium level (*p* = 0.002) was significantly higher, and serum potassium level (*p* = 0.026) was significantly lower at baseline in patients with hypernatremia (Table 1). We did not observe differences in urine examination results at baseline. When we calculated serum osmolality by sodium, blood urea nitrogen, and blood sugar levels, patients exhibiting hypernatremia showed significantly higher calculated serum osmolality (*p* = 0.012, Table 2). There were no differences in the doses of tolvaptan (7.5 ± 3.8 vs. 8.1 ± 2.5 mg/day, *p* = 0.269) and carperitide (0.025 ± 0.010 vs. 0.025 ± 0.06 μg/min, *p* = 0.835) between patients with and without hypernatremia.

**Table 1:** Baseline characteristics of patients on admission day with and without hypernatremia in the initial three days after tolvaptan treatment

	With hypernatremia	Without hypernatremia	*p* value
N (%)	22 (19)	95 (81)	
Age, years	78.5 ± 12.2	77.3 ± 11.3	0.661
Men, %	46	57	0.337
Body weight, kg	57.9 ± 13.8	58.6 ± 20.3	0.850
Body mass index, kg/m^2^	23.9 ± 6.3	23,4 ± 4.0	0.633
LVEF, %	44 ± 18	45 ± 19	0.869
Systolic blood pressure, mmHg	149 ± 21	137 ± 25	0.045
Diastolic blood pressure, mmHg	90 ± 20	78 ± 17	0.004
Heart rate, beats/min	95 ± 24	87 ± 23	0.152
NYHA, class			
I or II, %	77	55	0.089
III or IV, %	23	45	
Medical history			
Diabetes mellitus, %	36	42	0.623
Hypertension, %	73	64	0.452
Dyslipidemia, %	32	37	0.661
Atrial fibrillation, %	55	43	0.338
Coronary artery disease, %	23	25	0.806
Valvular disease, %	32	27	0.679
Cardiomyopathy, %	5	11	0.391
Medications before admission			
ACEI/ARB, %	41	39	0.867
β-blocker, %	55	40	0.217
Ca channel blocker, %	50	39	0.347
Loop diuretics, %	45	53	0.548
MRA, %	23	13	0.230
Thiazide, %	0	7	0.192
Laboratory data at baseline			
BNP, pg/mL	1043 ± 758	1127 ± 992	0.711
Hematocrit, %	35.3 ± 8.2	34.7 ± 7.5	0.722
Albumin, g/dL	3.9 ± 0.5	3.7 ± 0.5	0.182
Blood urea nitrogen, mg/dL	27.2 ± 19.8	27.5 ± 14.4	0.931
Serum creatinine, mg/dL	1.8 ± 2.3	1.3 ± 0.8	0.110
eGFR, mL/min/1.73m^2^	44.6 ± 20.9	46.6 ± 18.6	0.654
Uric acid, mg/dL	6.5 ± 2.4	6.4 ± 2.3	0.817
Serum sodium, mEq/L	143 ± 3	140 ± 4	0.002
Serum potassium, mEq/L	3.9 ± 0.5	4.3 ± 0.6	0.026
Blood sugar, mg/dL	145 ± 75	141 ± 57	0.812
Serum osmolality, mOsm/L	295 ± 9	291 ± 11	0.139
Hormone at baseline			
PRA, ng/mL/h	1.0 ± 1.5	2.7 ± 4.7	0.204
PAC, pg/mL	93 ± 108	117 ± 170	0.631
Adrenaline, pg/mL	75 ± 138	58 ± 133	0.690
Noradrenaline, pg/mL	1059 ± 723	709 ± 985	0.234
Dopamine, pg/mL	35 ± 27	47 ± 140	0.753
Urine examination at baseline			
Urine urea nitrogen, mg/dL	478 ± 397	478 ± 405	0.999
Urine creatinine, mg/dL	71.3 ± 64.9	86.0 ± 90.8	0.579
Urine sodium, mEq/L	84.4 ± 42.8	91.9 ± 50.1	0.546
Urine potassium, mEq/L	27.0 ± 18.4	29.7 ± 26.3	0.733
Urine osmolality, mOsm/L	450 ± 182	432 ± 188	0.707

*NYHA* New York Heart Association, *ACEI* angiotensin-converting enzyme inhibitor, *ARB* angiotensin receptor blocker, *MRA* mineralocorticoid receptor antagonist, *BNP* brain natriuretic peptide, *eGFR* estimated glomerular filtration rate, *PRA* plasma renin activity, *PAC* plasma aldosterone concentration

**Table 2:** Calculated parameters at baseline in patients with and without hypernatremia

	With hypernatremia	Without hypernatremia	*p* value
BUN/Cr	19.2 ± 7.6	22.5 ± 7.7	0.074
PAC/PRA	144 ± 144	142 ± 158	0.969
C-serum osmolality, mOsm/L	304 ± 7	298 ± 10	0.012
U-Osm/S-Osm	1.5 ± 0.6	1.5 ± 0.7	0.838
FENa, %	2.3 ± 2.5	3.3 ± 4.8	0.434
FEUN, %	36.8 ± 12.9	34.8 ± 12.6	0.623
FEK, %	18.1 ± 18.3	15.1 ± 10.4	0.442
TTKG	4.3 ± 1.7	4.3 ± 2.0	0.995

C-Serum osmolality = 2 × Na + BUN/2.8 + blood sugar/18; FENa = (U-Na × S-Cr)/(U-Cr × S-Na) × 100; FEUN = (U-UN × S-Cr)/(U-Cr × S-UN) × 100; FEK = (U-K × S-Cr)/(U-Cr × S-K) × 100; TTKG = {U-K/(U-Osm/S-Osm)}/S-K

*U-* urine-, *S-* serum-, *C-* calculated, *BUN/Cr* ratio of blood urea nitrogen to serum creatinine, *PAC/PRA* ratio of plasma aldosterone concentration to plasma renin activity, *U-Osm/S-Osm* ratio of urine osmolality to serum osmolality, *FENa* fractional excretion of sodium, *FEUN* fractional excretion of urea nitrogen, *FEK* fractional excretion of potassium, *TTKG* trans-tubular K gradient

**Fig. 1:**
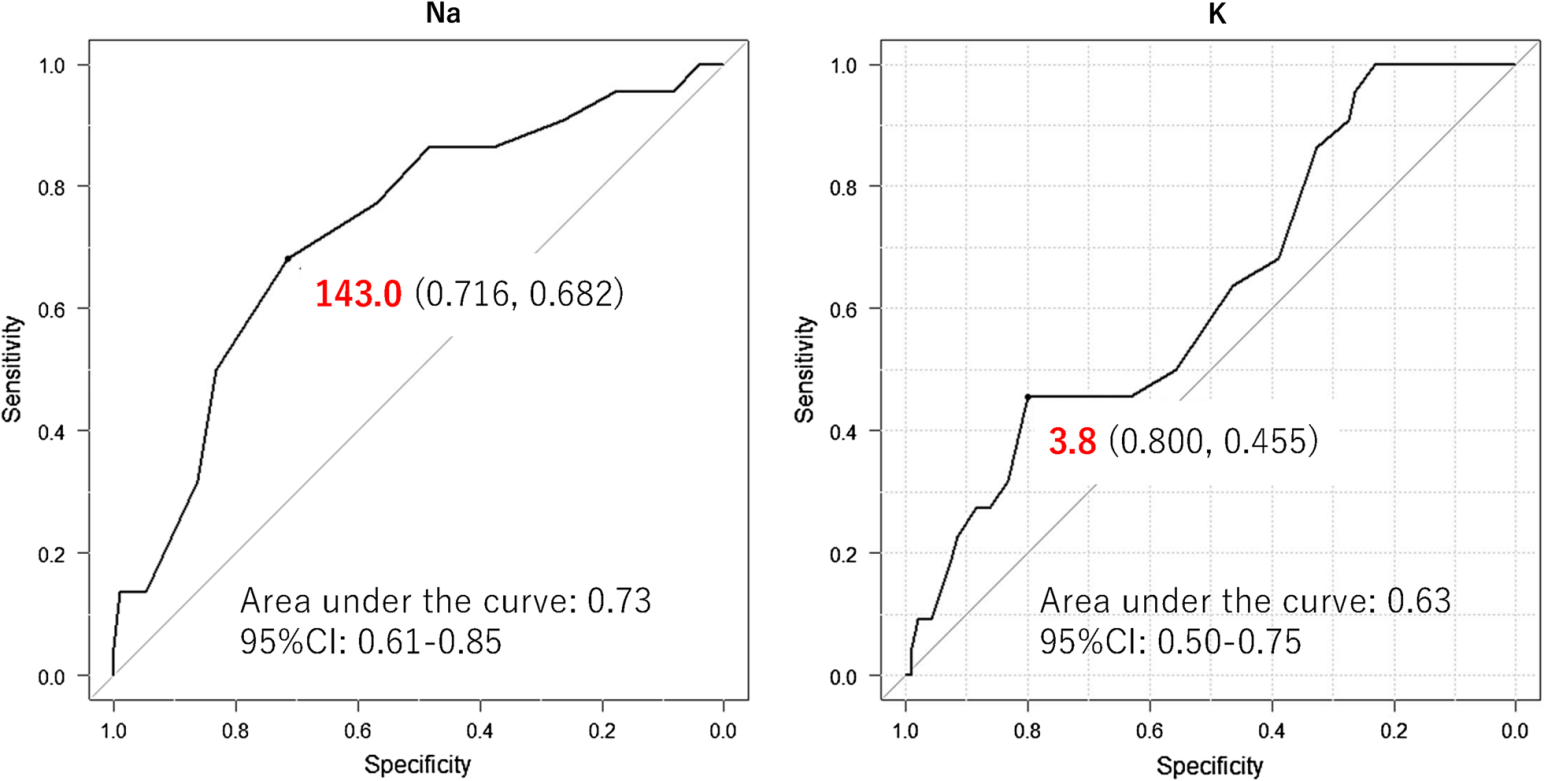
The cutoff serum sodium and potassium levels at baseline by receiver operating characteristic curve analysis in patients with decompensated heart failure with hypernatremia in the initial 3 days of hospitalization after tolvaptan administration in addition to standard therapy, including carperitide infusion

Regarding water balance calculated using the equation of (urine volume—water intake), dehydration obviously occurred during the first hospitalization day in patients with hypernatremia (*p* = 0.036, Table 3). In the multivariate regression analysis using significant factors observed in the univariate analysis, hypernatremia in the initial three days of hospitalization was independently associated with low serum potassium level (*p* = 0.034, Table 4). The cutoff serum potassium level at baseline was 3.8 mEq/L by the receiver operating characteristic curve analysis (Fig. 1).

**Table 3:** Water balance in patients with and without hypernatremia

	With hypernatremia	Without hypernatremia	*p* value
Total urine volume (BL to day 3), mL	10,610 ± 5327	8816 ± 4062	0.096
Total water intake (BL to day 3), mL	3530 ± 1643	2799 ± 1591	0.089
Urine volume—water intake, mL			
From BL to day 1	2188 ± 1583	1507 ± 1189	0.036
From days 1 to 2	1978 ± 1589	1700 ± 1480	0.465
From days 2 to 3	1681 ± 2051	1389 ± 1221	0.427

*BL* baseline

**Table 4:** Multivariate regression analysis of factors predicting hypernatremia

	OR	95% CI	*p* value
Systolic blood pressure, mmHg	0.99	0.965–1.020	0.678
Diastolic blood pressure, mmHg	1.04	0.994–1.080	0.094
Serum sodium level, mEq/L	1.14	0.878–1.480	0.326
Serum potassium level, mEq/L	0.28	0.085–0.907	0.034
C-serum osmolality, mOsm/L	1.09	0.981–1.210	0.109
Total urine volume			
Water intake (BL to day 1), mL	1.00	1.000–1.000	0.095

*OR* odds ratio, *CI* confidence interval, *C* calculated, *BL* base line

### Characteristics of patients with hypernatremia in those with low potassium level at baseline

There were no significant differences in the renin activity and aldosterone level and medications with loop diuretics, angiotensin-converting enzyme inhibitors/angiotensin receptor blockers, and aldosterone antagonists between patients with serum potassium level ≤ 3.8 mEq/L at baseline with and without hypernatremia (Table 5). However, patients with hypernatremia exhibited significantly higher diastolic pressure on admission day (*p* = 0.004) among those with serum potassium level ≤ 3.8 mEq/L (Table 5). The ratio of aldosterone level to renin activity tended to be high in patients with hypokalemia with hypernatremia.

**Table 5:** Baseline characteristics of patients whose baseline potassium level ≤ 3.8 mEq/L stratified based on the presence or absence of hypernatremia

	With hypernatremia (n = 10)	Without hypernatremia (n = 19)	*p* value
Serum sodium, mEq/L	144 ± 2	142 ± 2	0.080
ACEI/ARB, %	60	53	0.717
MRA, %	30	26	0.840
Loop diuretics, %	50	58	0.697
FENa, %	2.3 ± 2.1	3.7 ± 5.6	0.624
FEUN, %	42.0 ± 8.5	39.8 ± 9.0	0.665
FEK, %	12.6 ± 7.2	14.7 ± 10.1	0.684
TTKG	3.6 ± 1.8	4.0 ± 1.6	0.677
PRA, ng/mL/h	0.5 ± 0.4	3.5 ± 5.9	0.241
PAC, pg/mL	57.3 ± 31.0	80.1 ± 53.6	0.353
PAC/PRA	186 ± 211	114 ± 112	0.353
Systolic blood pressure, mmHg	157 ± 22	139 ± 22	0.050
Diastolic blood pressure, mmHg	97 ± 20	75 ± 17	0.004

*ACEI* angiotensin-converting enzyme inhibitor, *ARB* angiotensin receptor blocker, *MRA* mineralocorticoid receptor antagonist, *FENa* fractional excretion of sodium, *FEUN* fractional excretion of urea nitrogen, *FEK* fractional excretion of potassium, *TTKG* trans-tubular K gradient, *PRA* plasma renin activity, *PAC* plasma aldosterone concentration, *PAC/PRA*, ratio of plasma aldosterone concentration to plasma renin activity

## Discussion

Hypernatremia in the initial three days of hospitalization after tolvaptan administration in addition to standard therapy, including carperitide infusion, in patients with ADHF was associated with low serum potassium level at baseline in the multivariate regression analysis. Among patients with serum potassium level ≤ 3.8 mEq/L, the cutoff value by receiver operating characteristic curve analysis, those with hypernatremia related to tolvaptan treatment showed significantly higher diastolic blood pressure on admission day.

### Tolvaptan and renin–angiotensin–aldosterone system (RAAS)

Secondary aldosteronism with HF and loop diuretic therapy may be attributed to hypokalemia [15]. Aldosterone stimulates sodium reabsorption and potassium excretion via Na + -K + ATPase in the renal tubules of these patients, leading to hypokalemia. However, tolvaptan inhibits angiotensin II-induced increases in aldosterone production via a V2 receptor-independent pathway in vitro [16]. Furthermore, treatment with tolvaptan plus natriuretic peptide does not activate RAAS [17] and prevents an increase in aldosterone levels compared to that with natriuretic peptide only [18]. In patients with hypokalemia at baseline, those with hypernatremia exhibited higher diastolic blood pressure, although there was no difference in medications with angiotensin-converting enzyme inhibitors/angiotensin II receptor blockers, aldosterone antagonists, and loop diuretics in those without hypernatremia in this study. The ratio of aldosterone level to renin activity tended to be higher in patients with hypokalemia with hypernatremia. These results suggest that the inhibitory effects of RAAS by RAAS inhibitor treatment were less or breakthrough phenomena of RAAS occurred in patients with hypokalemia with hypernatremia than in those without hypernatremia.

There are individual differences in the inhibitory extents of RAAS by RAAS inhibitor treatment, and the frequency of use of aldosterone blockade (approximately 20%) was lower in this study compared to those in other studies (approximately 40%) [11, 12]. The use of loop diuretics results in the inhibition of sodium reabsorption, but aldosterone blockade may be insufficient in patients with hypokalemia despite RAAS inhibitor treatment in consideration with higher blood pressure. Moreover, hypokalemia reduces urine concentration and induces an increase in urine volume, thus resulting in hypernatremia in addition to the effect of tolvaptan. These findings may indicate the pathophysiologically more severe state of HF in patients with hypokalemia with hypernatremia, which could be clarified by a further study examining prognosis in these patients.

It is well known that tolvaptan can decrease body weight and increase the sodium level in patients who are with ADHF [19]. We used the criteria of hypernatremia as sodium level ≥ 148 mEq/L (out of normal range in our hospital) in the initial three days of hospitalization, which was different from that in the previous study showing the risk factors for tolvaptan-induced hypernatremia (≥ 147 mEq/L [10]; ≥ 150 mEq/L [11, 12]). The incidence of hypernatremia was higher (19%) in this study than those in previous studies, resulting from the threshold of hypernatremia [11, 12] or included patients with liver cirrhosis (0.2%) [20]. In patients with liver cirrhosis, tolvaptan-induced hypernatremia was not related to hypokalemia, possibly because almost all patients with liver cirrhosis were administered spironolactone [10]. These findings strongly suggest that aldosterone-related factors may be involved in hypernatremia and hypokalemia of patients treated with tolvaptan. The combined use of tolvaptan and adequate RAAS inhibitors may be recommended to prevent hypernatremia in loop diuretic-refractory ADHF.

### Limitations

Some limitations are to be noted in this study: It is a single-center study; a study showing additive effect of tolvaptan in association with standard therapy, including carperitide infusion, in patients with ADHF; and not a dose-finding study. The routine use of carperitide is not recommended as a first-line vasodilator for elderly patients with ADHF [21]. Although urine examination result, such as urine osmolality, was used to predict response to tolvaptan [22], we did not observe differences in urine factors, such as urine osmolality and urine sodium/creatinine ratio, between patients with and without hypernatremia in this study. Some important clinical data such as echocardiographic indices were lacking for better, cautious understanding of the study results.

## Conclusion

In tolvaptan treatment combined with standard therapy in patients with ADHF, serum potassium level ≤ 3.8 mEq/L at baseline may be a determinant factor for the development of hypernatremia. Among patients with hypokalemia, those with higher diastolic blood pressure on admission may be carefully managed to prevent hypernatremia, possibly because of the involvement of aldosterone-related factors.

## Data Availability

The raw data may be made available upon reasonable request from the corresponding author.

## Abbreviations

ADHF: Acute decompensated heart failure
BNP: Brain natriutetic peptide
HF: Heart failure
RAAS: Renin-angiotensin-aldosterone system
UCr: Urine creatinine
UNa: Urine sodium
UUN: Urine urea nitrogen
